# Targeting stem-loop 1 of the SARS-CoV-2 5′ UTR to suppress viral translation and Nsp1 evasion

**DOI:** 10.1073/pnas.2117198119

**Published:** 2022-02-11

**Authors:** Setu M. Vora, Pietro Fontana, Tianyang Mao, Valerie Leger, Ying Zhang, Tian-Min Fu, Judy Lieberman, Lee Gehrke, Ming Shi, Longfei Wang, Akiko Iwasaki, Hao Wu

**Affiliations:** ^a^Department of Biological Chemistry and Molecular Pharmacology, Harvard Medical School, Boston, MA 02115;; ^b^Program in Cellular and Molecular Medicine, Boston Children’s Hospital, Boston, MA 02115;; ^c^Department of Immunobiology, Yale University School of Medicine, New Haven, CT 06520;; ^d^Institute for Medical Engineering and Science, Massachusetts Institute of Technology, Cambridge, MA 02139;; ^e^Department of Pediatrics, Harvard Medical School, Boston, MA 02115;; ^f^Department of Biological Chemistry and Pharmacology, The Ohio State University, Columbus, OH 43210;; ^g^The Ohio State University Comprehensive Cancer Center, The Ohio State University, Columbus, OH 43210;; ^h^Department of Microbiology, Blavatnik Institute, Harvard Medical School, MA 02115;; ^i^School of Life Science and Technology, Harbin Institute of Technology, Harbin 150001, China;; ^j^School of Pharmaceutical Sciences, Wuhan University, Wuhan 430071, China;; ^k^HHMI, Chevy Chase, MD 20815

**Keywords:** SARS-CoV-2, therapeutic, translation

## Abstract

SARS-CoV-2 is a highly pathogenic virus that evades antiviral immunity by interfering with host protein synthesis, mRNA stability, and protein trafficking. The SARS-CoV-2 nonstructural protein 1 (Nsp1) uses its C-terminal domain to block the messenger RNA (mRNA) entry channel of the 40S ribosome to inhibit host protein synthesis. However, how SARS-CoV-2 circumvents Nsp1-mediated suppression for viral protein synthesis and if the mechanism can be targeted therapeutically remain unclear. Here, we show that N- and C-terminal domains of Nsp1 coordinate to drive a tuned ratio of viral to host translation, likely to maintain a certain level of host fitness while maximizing replication. We reveal that the stem-loop 1 (SL1) region of the SARS-CoV-2 5′ untranslated region (5′ UTR) is necessary and sufficient to evade Nsp1-mediated translational suppression. Targeting SL1 with locked nucleic acid antisense oligonucleotides inhibits viral translation and makes SARS-CoV-2 5′ UTR vulnerable to Nsp1 suppression, hindering viral replication in vitro at a nanomolar concentration, as well as providing protection against SARS-CoV-2–induced lethality in transgenic mice expressing human ACE2. Thus, SL1 allows Nsp1 to switch infected cells from host to SARS-CoV-2 translation, presenting a therapeutic target against COVID-19 that is conserved among immune-evasive variants. This unique strategy of unleashing a virus’ own virulence mechanism against itself could force a critical trade-off between drug resistance and pathogenicity.

## Significance

The COVID-19 pandemic and the ever-evolving variants of SARS-CoV-2 are taking a toll on human health. Despite the successful rollout of vaccines, effective therapies are still urgently needed. Our studies here showing that Nsp1 selectively blocks translation of host but not viral proteins by proper coordination of its N- and C-terminal domains to advance our understanding on SARS-CoV-2 pathogenesis. Our finding that stem-loop 1, a highly conserved sequence in the SARS-CoV-2 5′ UTR, is necessary and sufficient for bypassing Nsp1-mediated shutdown led to the design of antisense oligonucleotides targeting this sequence that make viral translation susceptible to Nsp1 shutdown, interfere with viral replication, and protect SARS-CoV-2–infected mice. This strategy of turning SARS-CoV-2’s own virulence against itself could be harnessed therapeutically.

Severe acute respiratory syndrome coronavirus 2 (SARS-CoV-2), the causative agent of the infectious disease COVID-19, is a highly contagious and deadly virus with fast person-to-person transmission and potent pathogenicity (1, 2). It is an enveloped, single-stranded betacoronavirus that contains a positive-sense RNA genome of about 29.9 kb (3–5). The SARS-CoV-2 genome codes for two large overlapping open reading frames (ORF1a and ORF1b) and a variety of structural and nonstructural accessory proteins (6). Upon infection, the polyproteins ORF1a and ORF1b are synthesized by host machinery and proteolytically cleaved into 16 mature nonstructural proteins, namely Nsp1 to Nsp16 (1, 6, 7).

Nsp1 is a critical virulence factor of coronaviruses and plays key roles in suppressing host gene expression, which facilitates viral replication and immune evasion, presumably by repurposing the host translational machinery for viral production and preventing the induction of type I interferons (IFNs) (8–11). It has been shown that SARS-CoV Nsp1 effectively suppresses the translation of host messenger RNAs (mRNAs) by directly binding to the 40S small ribosomal subunit (12, 13). Recent cryoelectron microscopy (cryo-EM) structures of SARS-CoV-2 Nsp1 indeed reveal the binding of its C-terminal domain (CT) to the mRNA entry channel of the 40S subunit (14–17), which contributes to blocking translation. These structural data are further supported by experiments demonstrating that Nsp1 binding to the 40S ribosome requires an open-head conformation induced by core elongation initiation factors and that Nsp1 cannot bind to a 40S with an mRNA already occupying the entry channel (18).

Besides directly inhibiting mRNA translation, Nsp1 has also been shown to reduce the available pool of host cytosolic mRNAs by both promoting their degradation and inhibiting their nuclear export (19–21). Mutants of Nsp1 that disrupt ribosome binding also abolish mRNA degradation, suggesting that the degradation is likely downstream of the Nsp1 translational block, and these two processes likely synergize to shut off host protein expression (12, 22).

Previous studies on SARS-CoV and SARS-CoV-2 have implicated stem-loop 1 (SL1) in the leader region of the 5′ untranslated region (5′ UTR) in protecting the virus against Nsp1-mediated mRNA translation inhibition (9, 17, 22, 23). However, how SARS-CoV-2 overcomes Nsp1-mediated translation suppression for its replication and whether this mechanism can be targeted for therapeutic intervention remain open questions. Here, we show that SARS-CoV-2 depends on SL1 to escape Nsp1 suppression to effectively switch the translational machinery from synthesizing host proteins to making viral proteins, and that both the CT and the N-terminal domain (NT) are required for the transition from host to viral translation. The latter is supported by complementary experiments in a study released while this paper was in preparation (24). We further show that SL1 can be targeted by locked nucleic acid (LNA) antisense oligonucleotides to prevent the SARS-CoV-2 5′ UTR from evading its own translational suppression to potently inhibit viral replication.

## Results

### SARS-CoV-2 5′ UTR Mediates Translation Despite the Presence of Nsp1.

**Fig. 1.**
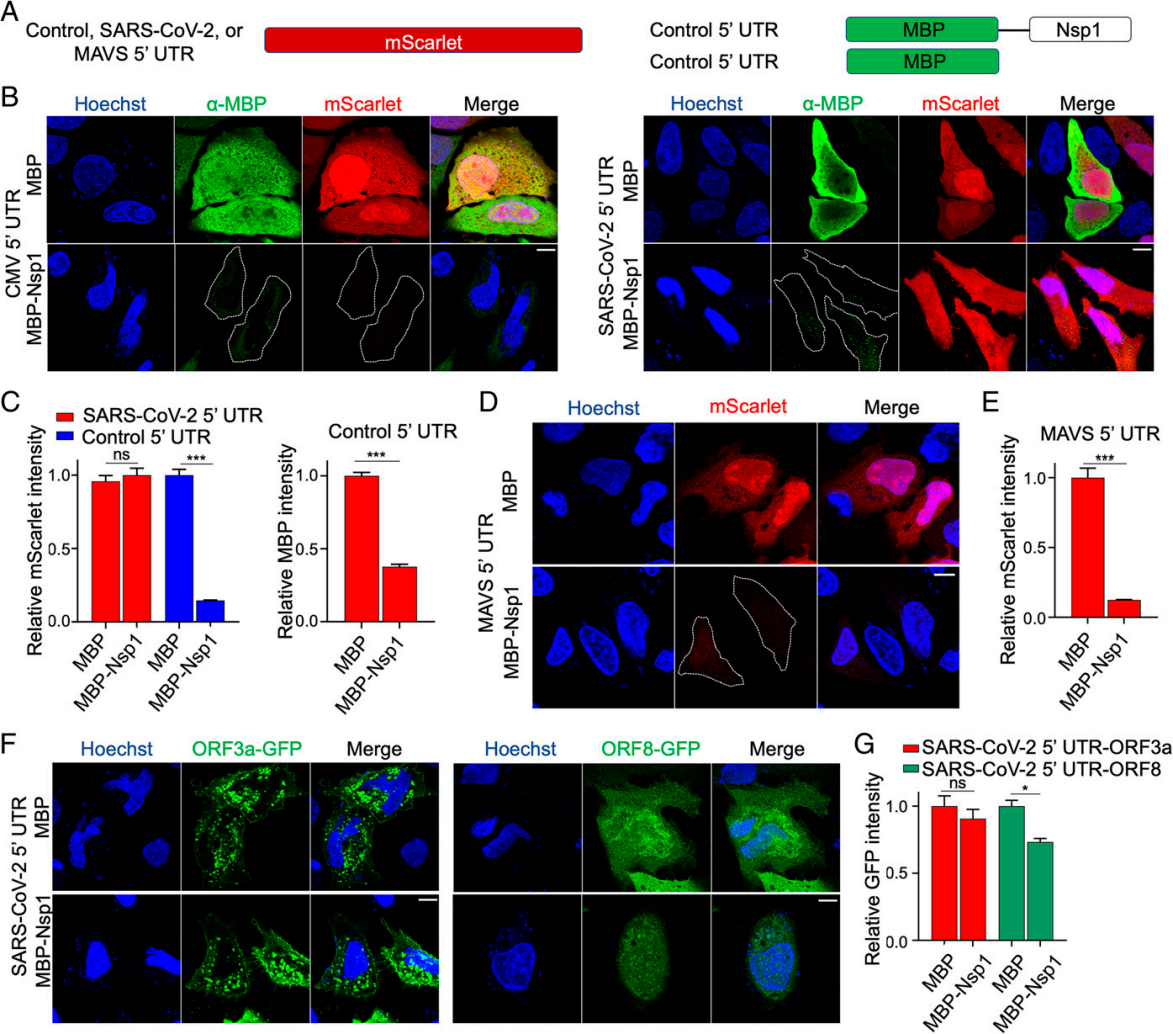
SARS-CoV-2 5′ UTR bypasses Nsp1-mediated inhibition of translation. (*A*) Schematic of translational reporters. The 5′ UTR sequences from control, MAVS, or SARS-CoV-2 were placed upstream of the mScarlet reporter (*Left*). MBP or MBP-Nsp1 (*Right*) were both downstream of the control 5′ UTR and were cotransfected along with each reporter plasmid. A CMV promoter was used to drive expression in all constructs. (*B*) Representative images of HeLa cells cotransfected with control 5′ UTR reporter or SARS-CoV-2 5′ UTR reporter and either MBP alone or MBP-Nsp1 and visualized for DNA by Hoechst (blue), MBP by indirect immunofluorescence (green), and mScarlet by in situ fluorescence (red). Successfully transfected cells difficult to visualize due to low intensity are outlined here and in other figures. (*C*) Quantification of relative mScarlet intensity of data corresponding to *B*. (*D*) Representative images of HeLa cells transfected with MAVs 5′ UTR reporter (red). (*E*) Quantification of relative mScarlet intensity in *D*. (*F*) HeLa cells transfected with either ORF3a-GFP (*Left*) or ORF8-GFP (*Right*) downstream of SARS-CoV-2 5′ UTR. (*G*) Quantification of relative GFP intensity in *F*. Error bars correspond to SEM except where otherwise noted. (Scale bars, 10 μm.) *P < 0.05, **P < 0.01, and ***P < 0.001, Student *t* test.

To investigate the function of SARS-CoV-2 Nsp1 in inhibiting mRNA translation, we cotransfected an mScarlet reporter construct with maltose binding protein (MBP)-tagged Nsp1 or the MBP control in HeLa cells, and imaged mScarlet fluorescence and anti-MBP immunofluorescence (Fig. 1 *A* and *B*). The mScarlet reporter used an expression vector that contains the cytomegalovirus (CMV) promoter and 5′ UTR and is commonly employed for mammalian cell expression (referred to as control reporter throughout the paper). The MBP and MBP-Nsp1 constructs also used the CMV promoter and 5′ UTR. Upon analysis of the control reporter, we found that mScarlet expression in MBP-Nsp1–transfected cells was reduced by over 7.1-fold (*P* < 0.001) compared to cells cotransfected with MBP-alone (Fig. 1 *B* and *C*). These data are consistent with previous reports indicating potent translational suppression by Nsp1 (14, 17).

On the other hand, when the 5′ UTR in the control mScarlet reporter was replaced by the SARS-CoV-2 5′ UTR (referred to as the CoV-2 reporter throughout the paper), no significant difference in mScarlet expression was observed upon coexpression with MBP-Nsp1 or MBP, indicating robust evasion of Nsp1-mediated translational suppression (Fig. 1 *A–C*). In contrast, in this same experiment, CMV 5′ UTR-controlled MBP-Nsp1 showed significantly lower expression than MBP alone (*P* < 0.001) (Fig. 1 *B* and *C*). Thus, expression of Nsp1 from this construct was likely self-limiting, yet still sufficient to inhibit translation of mRNAs with non–SARS-CoV-2 5′ UTR but not the reporter with SARS-CoV-2 5′ UTR. Since the CMV 5′ UTR is not representative of those in human mRNAs, we also generated a reporter containing the 5′ UTR from human mitochondrial antiviaral signaling protein (MAVS), an essential signaling effector responsible for certain virus-induced production of type I and III IFNs, including SARS-CoV-2 (25). This mScarlet reporter was also potently suppressed by MBP-Nsp1, which decreased its expression 8.1-fold relative to MBP alone (*P* < 0.001) (Fig. 1 *A*, *D*, and *E*), suggesting that Nsp1-mediated translational suppression of host mRNAs can contribute to disabling critical mediators of the antiviral IFN response.

To model subgenomic RNAs generated during discontinuous SARS-CoV-2 gene transcription, we tested constructs with SARS-CoV-2 5′ UTR upstream of either GFP-fused ORF3a or ORF8 (ORF3a-GFP or ORF8-GFP). ORF3a-GFP expression was not significantly decreased by Nsp1 coexpression, and ORF8-GFP showed only a 1.4-fold decrease (Fig. 1 *F* and *G*), which was modest relative to the 7.1-fold and 8.1-fold decrease seen with CMV 5′ UTR and MAVS 5′ UTR, respectively (Fig. 1 *C* and *E*). Together these data support that SARS-CoV-2 Nsp1 potently inhibits host protein translation and that SARS-CoV-2 5′ UTR allows evasion of Nsp1-mediated suppression.

### The SL1 of the 5′ UTR Is Necessary and Sufficient for Evasion of Nsp1-Mediated Translation Suppression.

**Fig. 2.**
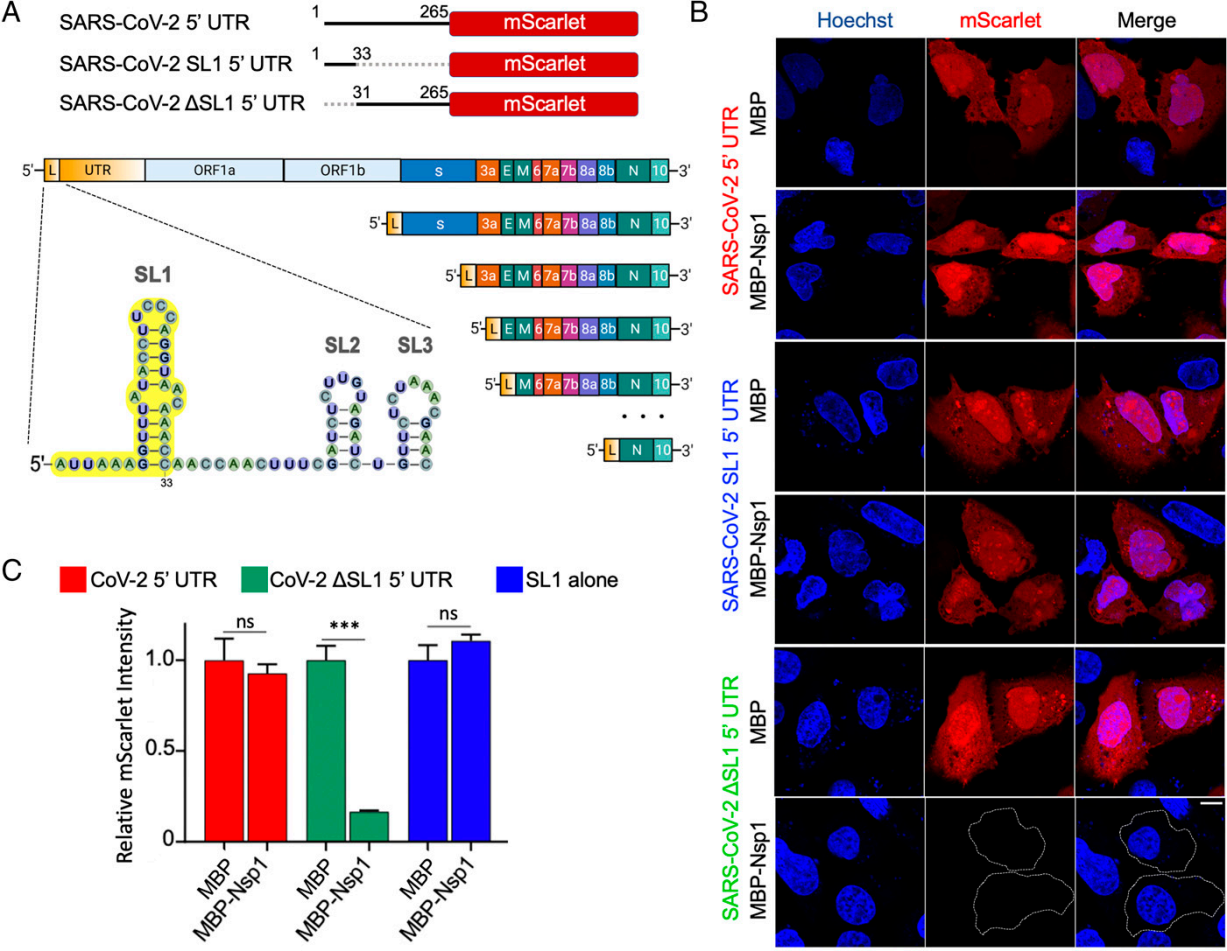
The SL1 stem-loop of the 5′ UTR is necessary and sufficient for evasion of Nsp1-mediated translation suppression. (*A*) Schematic representation of 5′ UTR, SL1 5′ UTR, and ΔSL1 5′ UTR placed upstream of mScarlet (*Upper*), and of SARS-CoV-2 leader sequence containing SL1 (yellow) along with its incorporation into subgenomic RNAs (*Lower*). (*B*) Representative images of HeLa cells cotransfected with SARS-CoV-2 5′ UTR reporter and either MBP-alone or MBP-Nsp1, and visualized for DNA by Hoechst (blue) and mScarlet by in situ fluorescence (red). (*C*) Quantification of relative mScarlet intensity of data corresponding to *B*. (Scale bars, 10 μm.) *P < 0.05, **P < 0.01, and ***P < 0.001, Student *t* test.

The 5′ UTR of coronaviruses comprises a number of stem-loop structures (*SI Appendix*, Fig. S1), among which SL1 has been shown to play critical roles in driving viral replication (12–14, 17, 26). For SARS-CoV-2, this conclusion should also make sense as the leader sequence driving all subgenomic RNAs is comprised of just SL1 to SL3 instead of the entire 5′ UTR (27, 28), highlighting the potential importance of SL1 in both viral replication and possibly in promoting evasion from translation suppression by Nsp1. To test the latter function of the SL1 sequence of SARS-CoV-2, we generated ΔSL1 and SL1-alone 5′ UTR mScarlet reporters (Fig. 2*A*). Compared with mScarlet translation in control cells cotransfected with MBP, the expression of SARS-CoV-2 ΔSL1 5′ UTR mScarlet reporter in MBP-Nsp1–transfected cells was 6.3-fold reduced (*P* < 0.001) (Fig. 2 *B* and *C*), a reduction similar to the control and MAVS 5′ UTR reporters above (Fig. 1 *C* and *E*). These data suggest that SL1 is completely required for evasion of Nsp1-mediated translation suppression. Interestingly, the reporter bearing only the SL1 sequence in its 5′ UTR was not significantly reduced upon coexpression with Nsp1, indicating that the SL1 sequence is both necessary and sufficient for evasion of Nsp1-mediated translation suppression in our experimental system (Fig. 2 *B* and *C*).

### Nsp1-CT and Nsp1-NT Are both Required for Optimal Host Suppression and SL1-Driven Bypass.

**Fig. 3.**
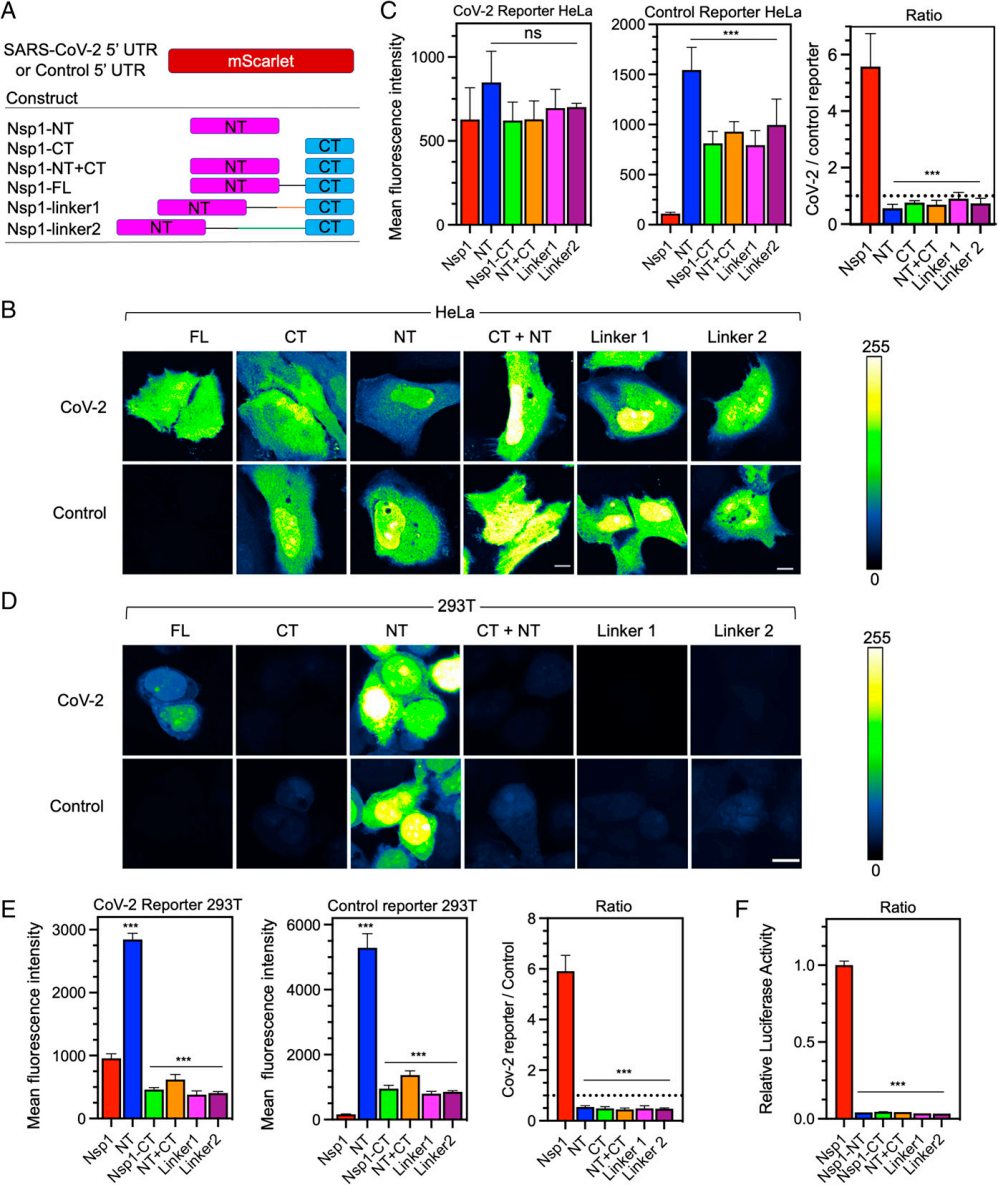
Nsp1 NT and CT cooperate to drive viral translation selectivity. (*A*) Schematic of coexpression system with CoV-2 or control reporter along with various fragments of Nsp1 (FL, NT, CT, NT+CT) or extended linker mutants (linker1, linker2). (*B*) mScarlet fluorescence intensity in HeLa cells cotransfected with either the CoV-2 (*Upper*) or control reporter (*Lower*) along with various mutants of Nsp1. Intensity values are fals- colored according to a scale (*Right*). (Scale bar, 10 μ.) (*C*) Quantification of fluorescent intensity in *B* of CoV-2 (*Left*) or control reporters (*Center*) and the ratio of CoV-2/Control (*Right*) with different Nsp1 mutants. (Dashed line marks ratio of 1). (*D*) mScarlet fluorescence intensity in 293T cells as in *B*. (*E*) Quantification of *D*. (*F*) Relative luciferase activity in 293T cells assay cotransfected with CoV-2 firefly luciferase and control *Renilla* luciferase reporters along with various Nsp1 mutants. The ratio of firefly/*Renilla* luciferase was normalized and plotted in three replicates. Error bars represent SD. (Scale bars, 10 μm.) *P < 0.05, **P < 0.01, and ***P < 0.001, Student *t* test.

Nsp1 is a 187-aa protein with a 128-aa NT and a 33-aa CT separated by a short linker region (Fig. 3*A*). Previous structures of the Nsp1−ribosome complex suggest that Nsp1-CT blocks mRNA entry to the ribosome and should be sufficient to inhibit protein translation (14, 16, 17). In order to probe the relative functions of these domains in suppressing host translation and allowing bypass by SARS-CoV-2 5′ UTR, we cotransfected HeLa cells with SARS-CoV-2 or control reporters along with Nsp1 full-length (FL), NT, CT, or both NT and CT driven from different constructs (NT+CT) (Fig. 3 *B* and *C*). None of these treatments significantly compromised CoV-2 5′ UTR reporter activity relative to FL Nsp1; however, NT, CT, and NT+CT less efficiently inhibited the control reporter mScarlet fluorescence intensity relative to FL Nsp1, with NT being the least effective (Fig. 3 *B* and *C* and *SI Appendix*, Fig. S2). We repeated this experiment in HEK293T cells and found that the CoV-2 5′ UTR reporter activity was significantly higher with coexpression of NT and significantly lower with CT in comparison with FL Nsp1, suggesting impaired evasion of CT-imposed translational block (Fig. 3 *D* and *E* and *SI Appendix*, Fig. S2). While FL Nsp1 most effectively suppressed the control reporter, the trend of suppression of the control reporter by NT, CT, or NT+CT mirrored that of the CoV-2 5′ UTR reporter (Fig. 3*E*).

These apparently different observations from HeLa versus 293T cell lines were intriguing, but when we looked at the ratio of the CoV-2 5′ UTR reporter to the control reporter fluorescence, we found a strikingly similar trend. FL Nsp1 coexpression led to a CoV-2/control fluorescence ratio of 5.6 ± 1.2 and 5.9 ± 0.62 for HeLa and 293T cells, respectively (Fig. 3 *C* and *E*). In both cell lines, coexpression of either CT or NT led to an ∼10-fold reduction in CoV-2/control fluorescence ratio, and coexpression of NT+CT from separate constructs reduced CoV-2/control ratio to a similar extent (Fig. 3 *C* and *E*). To further validate these observations, we utilized a luciferase reporter in 293T cells—which also relies on ratiometric normalization to a control reporter—and found a similar reduction in CoV-2 reporter translation selectivity by the different Nsp1 constructs (Fig. 3*F*). Collectively, these data suggest that NT and CT are both important for host translational suppression, which is consistent with a recent study (24), and that the NT is required for viral evasion of Nsp1-mediated translational suppression. In addition, Nsp1 may be tuned to control the ratio of viral/host translation rather than simply promoting high viral translation, perhaps to maintain some level of host fitness to allow viral replication.

### Correct Association and Spacing of NT+CT via the Nsp1 Linker Is Required for Function.

The fact that NT+CT expression from different constructs compromised the CoV-2/control ratio suggested a role for covalent association between the two domains via a linker. To test whether the length of the linker between the NT and CT in Nsp1 has any functional effect, we inserted an additional 20 residues (linker1) or 40 residues (linker2) at the Nsp1 linker region (Fig. 3*A*). Remarkably, the linker extensions dramatically reduced the ratio between CoV-2 and control reporter expression (*P* < 0.001) in both HeLa and 293T cell lines (Fig. 3 *B–E*). These data were also validated in the SARS-CoV-2 5′ UTR luciferase assay (Fig. 3*F*), suggesting that the NT and CT must somehow cooperate in a spatially specific manner to allow optimal suppression of host translation, and to permit the evasion of suppression on viral translation. Consistent with another report, we also observed lack of nuclear localization when visualizing Nsp1 FL, CT, linker1, and linker2 (19). NT alone, however, showed both nuclear and cytosolic localization, suggesting a role for CT and linker regions for sequestering Nsp1 in the cytosol (*SI Appendix*, Fig. S2).

Various naturally occurring mutations have been described in Nsp1 throughout the pandemic, including a 3-aa deletion in the Nsp1 linker region (Nsp1^ΔKSF^) detected in North America and Europe (29, 30). Given the importance of the Nsp1 linker length in regulating viral-to-host translation selectivity, we tested the function of this variant using our reporter assay. Although Nsp1^ΔKSF^ induced a small significant decrease in CoV-2 reporter activity, it more than doubled control reporter activity compared to WT Nsp1, and significantly reduced the CoV-2/control translation ratio (*P* < 0.001) (*SI Appendix*, Fig. S3). Thus, while lengthening the linker alters regulation of both viral and host translation, the shortened linker in this variant mainly compromised suppression of host translation. Together, these results suggest that the Nsp1 linker length is optimized to coordinate host translational suppression and bypass by SARS-CoV-2 5′ UTR, and that the Nsp1^ΔKSF^ mutant could be less virulent.

It has been recently determined that Nsp1 promotes degradation of host mRNAs whose translation is suppressed (20), which depends on R124, a key residue in the NT that is conserved in SARS-CoV (9, 17). To test whether this residue affects host translational shutdown, we coexpressed our control reporter with Nsp1^R124A^, which increased reporter activity by fivefold relative to control (*P* < 0.001). Interestingly, this mutation also led to a 1.5-fold decrease in CoV-2 reporter activity (*P* < 0.001) and significantly reduced the CoV-2/control ratio (*P* < 0.001) (*SI Appendix*, Fig. S3). These results suggest that mRNA degradation could indeed contribute to host shutdown and is consistent with the idea that it reduces the pool of available host mRNAs able to compete with SARS-CoV-2 RNA for ribosome association. It was reported by two other studies that R124A does not interfere with translational suppression of host mRNAs, but these studies relied on in vitro translation in cell extracts that are not optimized to recapitulate Nsp1-directed host mRNA degradation (18, 24).

### SL1 Antisense Oligos Selectively Target SARS-CoV-2 5′ UTR with Nanomolar Potency.

**Fig. 4.**
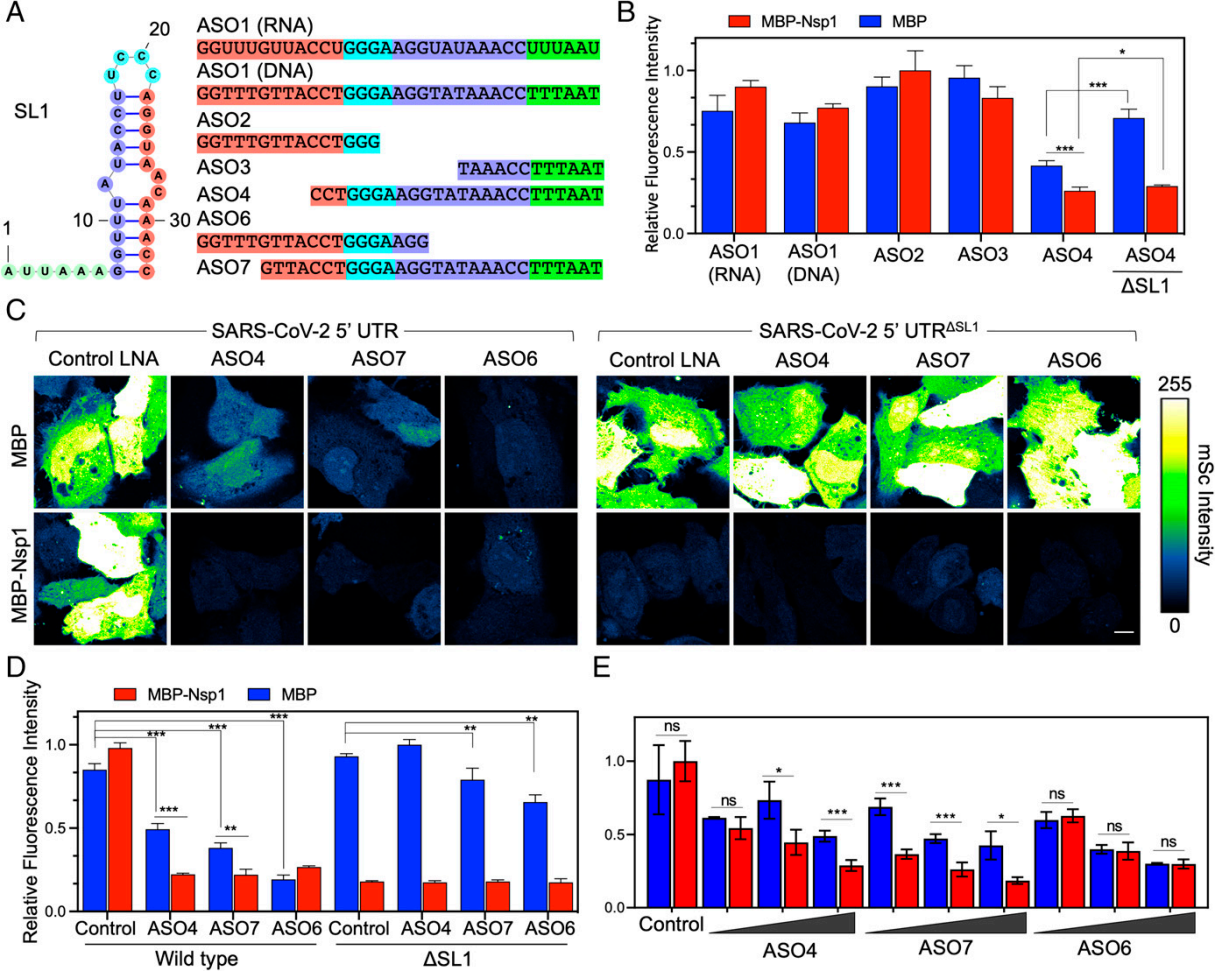
ASOs targeting SL1 renders the SARS-CoV-2 5′ UTR susceptible to Nsp1-mediated shutdown. (*A*) Schematic of SL1 region and various ASOs (which are all LNA mixmers unless otherwise noted). Complementary sequences between ASOs and SL1 are matched by color. (*B*) Initial screen of ASO activity. Each ASO was transfected at 50 nM with CoV-2 reporter along with either MBP-alone or MBP-Nsp1. Bar on far right indicates cotransfection with a reporter lacking SL1 as a control. (*C*) Images of HeLa cells transfected with 50 nM ASO4, -7, -6, or a control ASO along with CoV-2 or ΔSL1 reporter and either MBP or MBP-Nsp1. Intensity values are false-colored according to a scale (*Right*). (*D*) Quantification of *C*. (*E*) Dose–response assay of each ASO. Cells were transfected with CoV-2 reporter and MBP or MBP-Nsp1 as above. ASOs were transfected at either 25, 50, or 100 nM. (Scale bars 10 μm.) *P < 0.05, **P < 0.01, and ***P < 0.001, Student *t* test.

To suppress viral translation, we attempted to disrupt SL1’s function using antisense oligonucleotides (ASO). Since SL1 is sufficient for both viral translation and evasion of Nsp1-mediated translation suppression, ASOs targeting SL1 could represent novel therapeutic opportunities to effectively inhibit viral translation. SL1 starts right after the 5′ cap, and its structure is dynamically regulated during viral replication (31). The stem region of SL1 contains 10 Watson Crick base pairs with a bulge at the center (Fig. 4*A*) (32). We rationally designed different ASOs to hybridize with various regions of the SL1 and tested their activity against our CoV-2 5′ UTR reporter in the presence or absence of Nsp1.

In preliminary experiments, when transfected at 50 nM, neither DNA nor RNA anti-SL1 ASOs showed activity against CoV-2 5′ UTR in the presence or absence of Nsp1 (Fig. 4*B*). We then further designed ASOs with LNA mixmers targeting various regions of SL1 (Fig. 4*A*). ASO2 and ASO3 LNAs (short, ≤15 bases) targeted the 5′ and 3′ regions of SL1, respectively, but both failed to suppress SL1 activity (Fig. 4*B*). ASO4, a 24-base LNA against the 3′ region of SL1, successfully suppressed reporter activity on its own when cotransfected at 50 nM with the CoV-2 reporter and MBP alone (Fig. 4*B*). Interestingly, this suppression was further enhanced upon coexpression with Nsp1 (Fig. 4*B*), indicating successful inhibition of SL1-mediated evasion of Nsp1 translational shutdown. In the presence of Nsp1, suppression of CoV-2 5′ UTR reporter activity by ASO4 was even significantly lower when compared to the ΔSL1 reporter, demonstrating that ASO4 induces a complete loss-of-function of the SL1 sequence (Fig. 4*B*). We additionally designed two more LNA ASOs of similar lengths—ASO6 and ASO7—against the 5′ and 3′ regions of SL1, respectively. Like ASO4, both ASO6 and ASO7 suppressed the SARS-CoV-2 5′ UTR reporter on their own and showed relatively little activity against the same reporter lacking the SL1 sequence (Fig. 4 *C* and *D*). Interestingly, only ASO4 and ASO7, but not ASO6, synergized with Nsp1 to further suppress SARS-CoV-2 5′ UTR activity (Fig. 4 *C* and *D*). This tendency was consistent when the ASOs were tested over various concentrations (25, 50, and 100 nM) (Fig. 4*E*). Together, these data suggest that ASO4 and ASO7 suppress viral translation in at least two ways: 1) by making the 5′ UTR less efficient in driving viral translation, and 2) by interfering with the evasion of the 5′ UTR from Nsp1-mediated suppression.

### SL1 ASOs Inhibit SARS-CoV-2 Replication in Vero E6 Cells and Provide Significant Protection Against SARS-CoV-2–Induced Lethality in K18-hACE2 Mice.

**Fig. 5.**
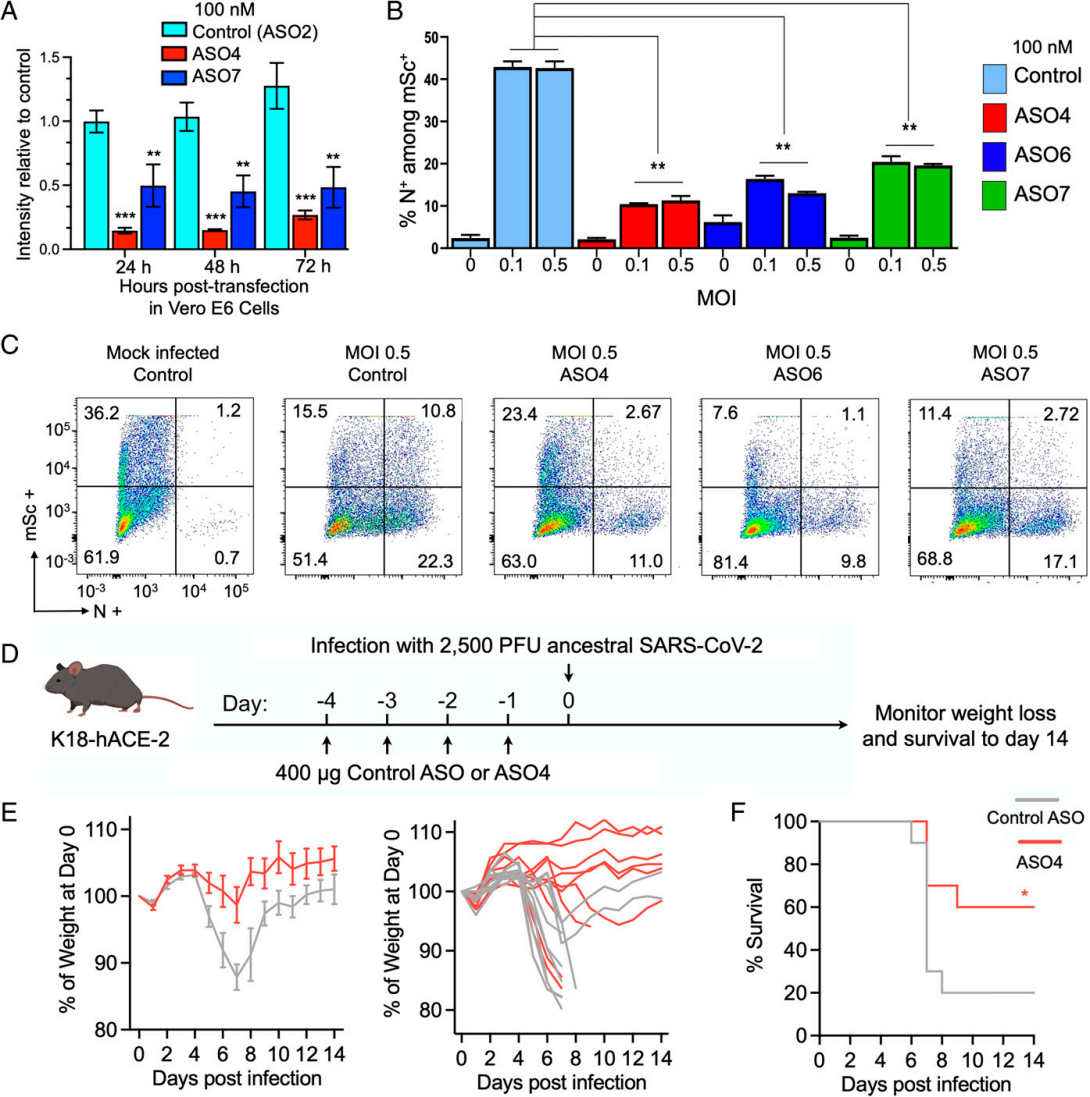
ASOs targeting SL1 produce stable loss of function to inhibit SARS-CoV-2 replication in vitro and ASO4 provides significant protection against SARS-CoV-2–induced lethality in K18-hACE2 mice. (*A*) Various ASOs along with CoV-2 reporter and MBP-Nsp1 were transiently transfected into Vero E6 cells and reporter intensity was measured daily over the course of 72 h, shown for each ASO (control, ASO4, and ASO7) at each time point. Since expression from transfected plasmids naturally changes over time, each datapoint was normalized to intensity of a parallel control where no ASO was included. (*B*) Percent of successfully transfected cells (marked by mScarlet [mSc] positivity) that were N^+^ by ASO treatment (color coded according to the legend on the right) at various MOIs. Error bars represent SD. (*A* and *B*) *P < 0.05, **P < 0.01, and ***P < 0.001, Student *t* test. (*C*) Nucleocapsid intensity plotted against mSc obtained by flow cytometry for each treatment (infected at MOI 0.5). Quadrants demarcate mSc^+^, N^+^ cells (top right quadrant), and the corresponding percentage of cells is listed in each corner. (*D*) Schematic for mouse infection experiment. K18-hACE-2 mice were treated with daily intranasally administered control ASO or ASO4 for 4 d following infection with 2,500 PFU of SARS-CoV-2 and monitored for weight loss and survival for 14 d. (*E*) Average percent weight loss over time for control ASO (gray) or ASO4 (red) after infection (*Left*) and individual weight loss trajectories (*Right*). (*F*) Survival curves over time for control ASO (gray) or ASO4 (red) after infection. *n* = 10 mice for each treatment.

To test whether anti-SL1 ASOs with activity in reporter assays could also inhibit viral replication in Vero E6 cells, we first confirmed the function of these ASOs in this cell line by cotransfecting them with SARS-CoV-2 5′ UTR reporter and Nsp1. When cotransfected at 100 nM, ASO4 and ASO7 gave a fivefold and twofold reduction, respectively, in reporter activity at 24 h (Fig. 5*A*). Importantly, this reduction in reporter activity persisted for at least 72 h, indicating that they retained function and stability upon transient delivery to cells (Fig. 5*A*). We then transfected Vero E6 cells with 100 nM of an ASO along with a control mScarlet reporter, followed by SARS-CoV-2 infection at either 0.1 or 0.5 multiplicity of infection (MOI). Cells were fixed 72 h postinfection and stained for nucleocapsid (N) to mark infected cells (Fig. 5 *B* and *C*). Roughly 37% of Vero E6 cells were mScarlet^+^ when transfected with control ASO and mock infected, which was used to indicate transfection efficiency (Fig. 5*C*). For either MOI 0.1 or 0.5, we observed an approximately fourfold reduction in N^+^ mScarlet^+^ cells with ASO4, approximately threefold reduction with ASO6, and an approximately twofold reduction with ASO7 when compared with control ASO (Fig. 5 *B* and *C*). Thus, our strategy for targeting SL1 with ASOs can successfully inhibit SARS-CoV-2 replication in vitro.

Given that ASO4 showed the highest protection against infection in Vero E6 cells, we further tested its antiviral efficacy in vivo using K18-hACE2 mice expressing the human angiotensin converting enzyme-2 (ACE2), the SARS-CoV-2 entry receptor. This model has been demonstrated to phenotypically recapitulate pathological and clinical features of COVID-19 (33, 34). K18-hACE2 mice were pretreated intranasally with 400 μg of naked ASO4 or a control ASO (scrambled) daily for 4 d before infection with 2,500 plaque forming units (PFU) of ancestral SARS-CoV-2 and monitored for 14 d (Fig. 5*D*). In the control ASO-treated group, 20% of mice survived infection and exhibited up to 12% weight reduction on average (Fig. 5 *E* and *F*). The ASO4-treated group showed a significant increase in survival (60%, *P* = 0.0477; log-rank test) with surviving animals showing minimal or no weight loss (Fig. 5 *E* and *F*). These results suggest that ASO4 can confer significant protection against SARS-CoV-2 when delivered to the respiratory tract prior to exposure and thus demonstrate the therapeutic efficacy of anti-SL1 targeting in vivo.

## Discussion

SARS-CoV-2 Nsp1 is a major virulence factor that suppresses host-gene expression and immune defense (9–11). The recently published cryo-EM structures (14, 17) showed that the helical hairpin at the CT of SARS-CoV-2 Nsp1 interacts with the 40S subunit of the ribosome to block mRNA entry. Here, we reveal that the NT also contributes to host translational suppression by coordinating with the CT, and that the NT and CT need to be covalently linked and correctly spaced to perform this function optimally. Insertion or deletion of linker residues between the NT and CT also compromises this function. We further found that SL1 in SARS-CoV-2 5′ UTR leads to evasion of Nsp1 suppression to allow viral translation, which also requires both the NT and CT. This finding is consistent with our analysis on the Nsp1 NT–CT linker deletion mutant as well as with other naturally isolated SARS-CoV-2 NT deletion mutants, which were associated with lower viral titers and less severe COVID-19 disease (30).

**Fig. 6.**
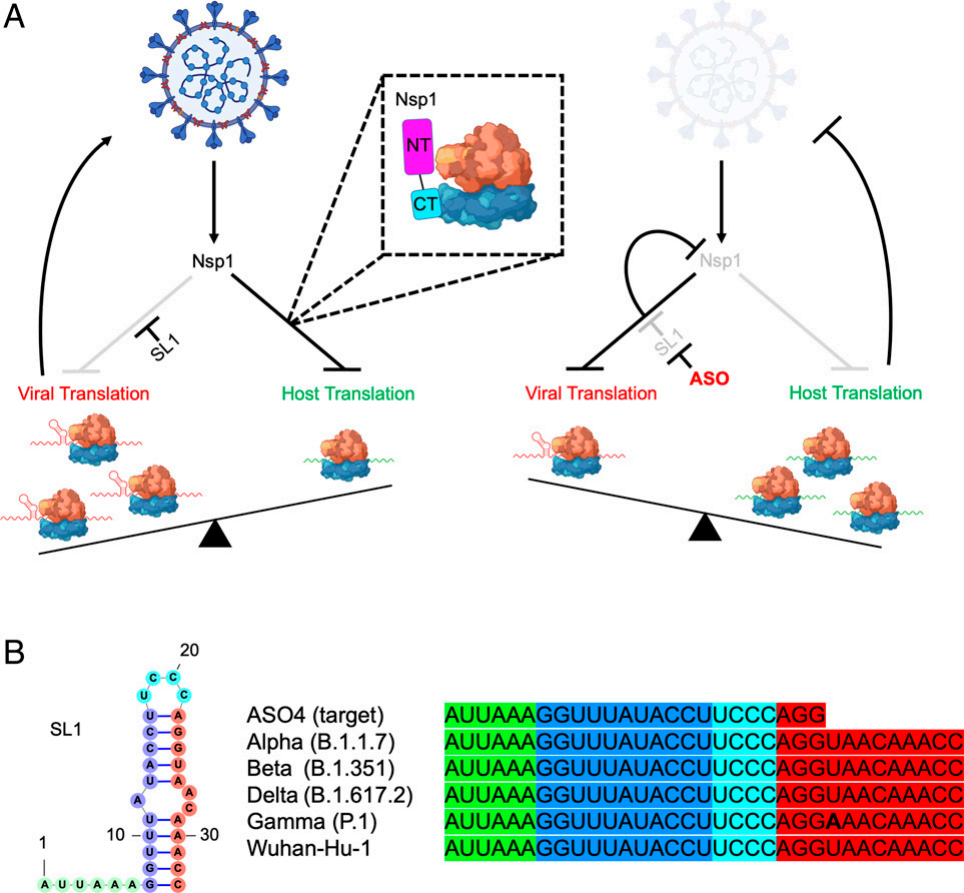
Model for Nsp1-driven viral translation selectivity and its disruption via ASO targeting of the highly conserved SL1 region. (*A*) Nsp1 shuts down host translation, mainly by blocking the mRNA entry channel of the 40S ribosome which ultimately results in host mRNA degradation. The SL1 region in SARS-CoV-2 5′ UTR allows evasion of translational suppression, leading to selective viral translation. Targeting SL1 via ASO makes SARS-CoV-2 5′ UTR vulnerable to Nsp1-mediated translation suppression, resulting in loss of translation of Nsp1 itself and restoration of host translation, allowing antiviral defense to more effectively halt viral replication. (*B*) Alignment of the ASO4 target sequence with SL1 sequences from SARS-CoV-2 variants of concern showing complete conservation of the sequence targeted by ASO4.

Our data show that the Nsp1 NT and CT coordinate to perform two functions: host translational suppression and bypass of this suppression by SARS-CoV-2 5′ UTR. The final outcome is that Nsp1 controls the viral-to-host translation ratio rather than simply promoting high viral translation, via both its NT and CT. Interestingly, while the effects of these two domains on absolute viral or host reporter activity differed by cell type, the ratio of viral-to-host translation was invariant. This tempered viral-to-host translation ratio imposed by Nsp1 could be optimal to maintain some level of host fitness to allow viral replication. It may also be tuned to produce the maximally allowable viral copy number that could still avoid triggering an IFN response. Thus, our studies show that coronaviruses like SARS-CoV-2 have evolved a clever strategy for controlling host translation machinery to support viral replication while counteracting the host cellular immune system (Fig. 6*A*).

A remaining question is whether the major driver of Nsp1-mediated host shutoff is depletion of host mRNAs or direct inhibition of translation. A recent study using ribosomal profiling and RNA sequencing found similar translation efficiencies of viral and host mRNAs during SARS-CoV-2 infection of Calu3 cells (20). One caveat is that these measurements were taken in late timepoints when viral RNAs had already started to enter the endomembrane system for packaging and would thus be shielded from the translation in the cytosol, potentially skewing the measurement of number of elongating transcripts per copy (20). A role for selective and direct translational block of host mRNAs is also supported by in vitro translation experiments (23, 24). Nonetheless it is likely that both mRNA degradation and inhibition of translation are mechanistically and functionally linked and both are major contributors to SARS-CoV-2 virulence.

The exact molecular basis for how Nsp1 NT coordinates with SL1 of SARS-CoV-2 5′ UTR to bypass the translation inhibition is still not yet clear. A previous study suggested that both FL and ΔCT Nsp1 directly bind the SL1 region of the CoV-2 5′ UTR by gel shift (35). However, this study was performed under low salt conditions, and we could not detect such interactions under physiological salt concentrations (*SI Appendix*, Fig. S4). Thus, the molecular details of NT coordination with SL1 remain elusive, and it is likely mediated by an indirect physical interaction.

While the classic model of eukaryotic translation initiation involves recognition of the 5′ cap by eIF4E followed by scanning of the UTR, recent studies revealed an alternative translation initiation regime that depends on the recruitment of the eIF3 complex by 5′ UTR stem-loop structures (36, 37). This complex is also engaged by stem-loop structures found in type 3 internal ribosome entry site elements, which depend on direct interaction with eIF3 to drive noncanonical translation in hepatitis c virus (37, 38). It is possible this alternative mode of translation initiation is mechanistically related to the Nsp1-SL1 axis during SARS-CoV-2 RNA translation and future studies will be critical for teasing out the functional interactions between Nsp1 and host proteins.

Our data also demonstrate that a single *cis*-acting element in the SARS-CoV-2 5′ UTR, the SL1 stem-loop, is both necessary and sufficient for evading Nsp1-mediated host shutdown and is thus a vulnerable therapeutic target for limiting SARS-CoV-2 replication (Fig. 6*A*). We revealed that SL1-targeting ASOs—including ASO4, ASO6, and ASO7—potently suppress SARS-CoV-2 5′ UTR reporters in HeLa and Vero E6 cells, and SARS-CoV-2 replication in Vero E6 cells at nanomolar concentrations. ASO4, the most effective ASO in vitro, provided significant protection against a lethal viral burden of SARS-CoV-2 upon preexposure intranasal administration in K18-hACE-2 mice, suggesting therapeutic activity in vivo. Given the poor efficiency of delivering nucleic acids to cells in the respiratory tract, optimizing delivery of ASO4 may further increase potency. Conjugation to cationic polymers or complexation with lipid particles or nanoparticles can boost uptake by cells in the respiratory tract and could increase protection against SARS-CoV-2 (39). Importantly, our ASOs were able to make SARS-CoV-2 vulnerable to its own mechanism of Nsp1-mediated host shutdown, a benchmark that could inform the development of future therapeutics targeting this critical mechanism. As we were preparing our paper, an independent preprint reported unbiased screening experiments that identified a promising ASO that is complementary to the 5′ UTR sequence, from the SL1-SL2 linker to the last 5 nucleotides of SL1, further underscoring its importance as a target (40) (*SI Appendix*, Fig. S4). However, it is unknown whether this ASO also sensitize SARS-CoV-2 to its own Nsp1-mediated translational suppression, like our ASO4 and ASO7.

Given that within the SL1 region there are no known single-nucleotide variants with >1% in frequency and no known mutations among variants of concern, this mechanism may represent a unique therapeutic target for immune-evasive, increasingly infectious strains that continue to emerge with the ongoing pandemic (Fig. 6*B*) (41). Early genomic data from the Omicron variant also indicate conservation (*SI Appendix*, Fig. S4). The recent appearance of variants, as well as the history of spontaneous resistance of viruses to conventional antiviral drugs—most notoriously to nucleoside inhibitors—suggests the need for new classes of antivirals (15, 42). The high conservation of both Nsp1 and SL1 and their requirement for viral replication suggest that SARS-CoV-2 mutants refractory to anti-SL1 ASO binding would be at a considerable selective disadvantage. This trade-off can be exploited by anti-SL1 therapy, which could thus represent a potent antiviral strategy whose evasion is evolutionarily constrained, requiring co-mutation of SL1 and Nsp1. More generally, our proof-of-principle in developing therapeutics to unleash a pathogen’s own virulence mechanism upon itself may represent an important strategy to avoid antiviral resistance in SARS-CoV-2 and could be expanded to other host–pathogen systems.

## Materials and Methods

### Plasmids and Transfection.

SARS-CoV-2 FL Nsp1 (1 to 180 aa), Nsp1-NT (1 to 127 aa), and Nsp1-CT (128 to 180 aa) were amplified from pDONR207 SARS-CoV-2 NSP1 (Addgene) by PCR and then cloned into pDB-His-MBP or BacMam pCMV-Dest plasmid. FL 265 nt 5′ UTR of SARS-CoV-2 was subcloned to replace the 5′ UTR of human CMV in the pLV-mScarlet vector using a Hifi one-step kit (Gibson Assembly, New England Biolabs). FL, SL1-alone, or ΔSL1 5′ UTR of SARS-CoV-2 were cloned into pLV-mScarlet vector or pGL3 basic vector. All constructs were verified by sequencing. Cells were transiently transfected with indicated plasmids using FuGENE Transfection Reagent (Promega) or Lipofectamine 2000 (Thermo Fisher Scientific) according to the manufacturer’s instructions. ASO transfection dosages correspond to the concentration at the time of lipid complex formation.

### Cell Culture.

HEK293T (ATCC CRL-3216, female), HeLa cells (ATCC CCL2, female), and Vero E6 (ATCC CRL1586) were purchased from the American Type Culture Collection, and Expi293 cells (female) were from ThermoFisher. Cells were cultured in Dulbecco’s Modified Eagle medium (Gibco) or Expi293 Expression Medium (Gibco) supplemented with 10% fetal bovine serum and 1% penicillin/streptomycin (Gibco) at 37 °C with 5% CO_2_.

### In Vitro Infection with SARS-CoV-2.

Vero E6 cells were seeded at ∼80% confluency in 24-well plates and cotransfected with 250 ng mScarlet-expressing plasmid and 100 nM of various ASOs before overnight incubation. The next day, the media was discarded, and cells were infected with SARS-CoV-2 (isolate USA-WA1/2020, ATCC NR-52281) at 0.1 or 0.5 MOI in low serum media and allowed to incubate 48 h. Cells were washed in PBS, fixed with CytoFix/CytoPerm solution (BD Sciences) for 30 min at room temperature, permeabilized with Perm/Wash Buffer (BD Sciences), blocked with 1% normal goat serum (Abcam, ab7481), and stained with antibodies to Nucleocapsid (Genetex GTX135357, 1:500) and Alexa 488-conjugated secondary antibody (Abcam ab150073; 1:1,000). Cells were analyzed by flow cytometry, and the percentages of mScarlet^+^, 488^+^, and double-positive cells were determined using FlowJo software (BD Sciences).

### Mice.

B6.Cg-Tg(K18-ACE2)2Prlmn/J (K18-hACE2) mice were purchased from the Jackson Laboratories and subsequently bred and housed at Yale University. Fourteen-week-old male mice were used for SARS-CoV-2 infection. All procedures used in this study complied with federal guidelines and the institutional policies of the Yale Institutional Animal Care and Use Committee.

### Intranasal ASO Treatment and SARS-CoV-2 Infection in Mice.

K18-hACE2 mice were anesthetized by intraperitoneal injection of ketamine (100 mg/kg body weight) and xylazine (10 mg/kg bodyweight) mixture diluted in 200 µL PBS per mouse. For intranasal ASO treatment, 50 µL of 400 μg ASO4 or control ASO were delivered intranasally daily for 4 d. On the fifth day, 50 µL of ancestral SARS-CoV-2 (USA-WA1/2020; BEI Resources) was delivered intranasally at 2,500 PFU per mouse (LD_100_). Following infection, weight loss and survival were monitored daily up to 14 d postinfection. Experiments involving SARS-CoV-2 infection were performed in a Biosafety Level 3 facility with approval from the Institutional Animal Care and Use Committee and Yale Environmental Health and Safety.

## Supplementary Material

Supplementary File

Supplementary File

## Data Availability

All study data are included in the main text and *SI Appendix*. Raw data is provided in Dataset S1.
