# Supplementary material for: Targeting stem-loop 1 of the SARS-CoV-2 5′ UTR to suppress viral translation and Nsp1 evasion
Source: Proc Natl Acad Sci U S A. 2022 Feb 11;119(9):e2117198119. doi: 10.1073/pnas.2117198119 (PMC8892331; doi:10.1073/pnas.2117198119)
Supplement: Supplementary File [file pnas.2117198119.sapp.pdf]

Supplementary Information for

Targeting Stem-loop 1 of the SARS-CoV-2 5'UTR to suppress viral translation and Nsp1 evasion

Setu M. Vora[a,b,1], Pietro Fontana[a,b,1], Tianyang Mao[c], Valerie Leger[d], Ying Zhang[b,e], Tian-Min Fu[a,b,f,g], Judy Lieberman[b,e], Lee Gehrke[d,h], Ming Shi[a,b,i,1,2], Longfei Wang[a,b,j,1,2], Akiko Iwasaki[c,k,2], and Hao Wu[a,b,2]

[a]Department of Biological Chemistry and Molecular Pharmacology, Harvard Medical School, Boston, MA 02115
[b]Program in Cellular and Molecular Medicine, Boston Children's Hospital, Boston, MA 02115
[c]Department of Immunobiology, Yale University School of Medicine, New Haven, CT 06520
[d]Institute for Medical Engineering and Science, Massachusetts Institute of Technology, Cambridge, MA 02139
[e]Department of Pediatrics, Harvard Medical School, Boston, MA 02115
[f]Department of Biological Chemistry and Pharmacology, The Ohio State University, Columbus, OH 43210
[g]The Ohio State University Comprehensive Cancer Center, The Ohio State University, Columbus, OH 43210
[h]Department of Microbiology, Blavatnik Institute, Harvard Medical School, MA 02115
[i]School of Life Science and Technology, Harbin Institute of Technology, Harbin 150001, China
[j]School of Pharmaceutical Sciences, Wuhan University, Wuhan 430071, China
[k]HHMI, Chevy Chase, MD 20815

[1]S.M.V., P.F., M.S. and L.W. contributed equally to this work.
[2]To whom correspondence may be addressed. Email: shiming@hit.edu.cn, wanglf@whu.edu.cn, akiko.iwasaki@yale.edu, or wu@crystal.harvard.edu

This PDF file includes:

- Supplementary Methods
- Figures S1 to S4
- Supplemental references

Other supplementary materials for this manuscript include the following:

- Dataset S1

Supplementary Information Text

Methods

Immunofluorescence and Quantitative Microscopy. To visualize the expression of mScarlet and MBP-tagged SARS-CoV-2 Nsp1 (WT, CT, NT, Nsp1-linker1 and Nsp1-linker2), transfected HeLa or 293T cells were fixed with 4% formaldehyde for 10 minutes at room temperature, permeabilized with PBS + 0.25% Triton X-100, and blocked with 3% BSA. Cells were stained with mouse anti-MBP (NEB, 1:500), followed by washing and subsequent incubation with goat anti-mouse IgG, Alexa Fluor 488 (1:1000, Invitrogen) as well as Hoechst 33342 counterstain (Immunochemistry). Cells were transfected in glass bottom 96 well plates (3-5 replicate wells per treatment) followed by imaging using an ImageXpress Micro Confocal (Molecular Devices) using a 20X objective (S Plan Fluor, NA=0.45). Multiple sites in each well were imaged to ensure large sample size (2-9, depending on instrument availability; each site contained ~100-200 cells). To obtain mean fluorescence intensity values, image data were analysed and quantified using Image J (NIH) or ImageXpress software (Molecular Devices). Representative images from Figures 1-4 were collected on an Olympus Fluoview FV1000 point scanning confocal microscope using a 60X UPlan S Apo water immersion objective (NA=1.2). For image data that displayed a large range of intensity values such as in Figures 3 and 4, images were contrast-adjusted equally between treatments, transformed to 8-bit and displayed using the 'Green Fire Blue' color scale in ImageJ.

Quantification and Data Analysis. Transfection experiments for microscopy were performed using at least 3 replicates in separate wells. The luciferase assay in Figure 3F was done on lysate samples pooled from 2 wells of replicates per treatment, and 3 separate measurements were taken for each lysate sample. Transfection-infection experiments in Figure 5E which were done in duplicate in 24 well plates. All graphs were plotted and analysed with GraphPad Prism 5 software. $p > 0.05$ was considered statistically not significant, and the following denotations were used: ***$p < 0.001$, **$p < 0.01$ and *$p < 0.05$. Sample size for each experiment corresponds to least 3 replicates except for Figure 5D which used two replicates. Sequence alignment in Figure 6C was carried out using Geneious Prime (Biomatters, Ltd.). Genomic RNA sequences for Wuhan-Hu-1, Beta (B.1.351), and Gamma (P.1) variants were provided by Genbank NCBI (NIH), and genomic RNA sequences for Alpha (B.1.1.7) and Delta (B.1.617.2) variants were provided by the GISAID database and correspond to the Accession IDs EPI_ISL_1501138 and EPI_ISL_2029113, respectively (SI ref 1). Predicted RNA structure of SARS-CoV-2 5'UTR shown in Figure S1 was carried out using RNAfold (University of Vienna) and was validated by comparison with a previously published RNA structure of the SARS-CoV-2 5'UTR (SI ref 2).

Luciferase Assay. Luciferase assays were performed as previously described (SI ref 3). Briefly, HEK293T or HeLa cells were transfected with luciferase and renilla reporter plasmids along with various Nsp1 constructs using Lipofectamine 2000 (Invitrogen). Luciferase assays were performed using the Dual Luciferase Assay System (Promega). Luciferase activity was measured using a CytoFluorplate 4000 Luminescence Microplate Reader (ABI).

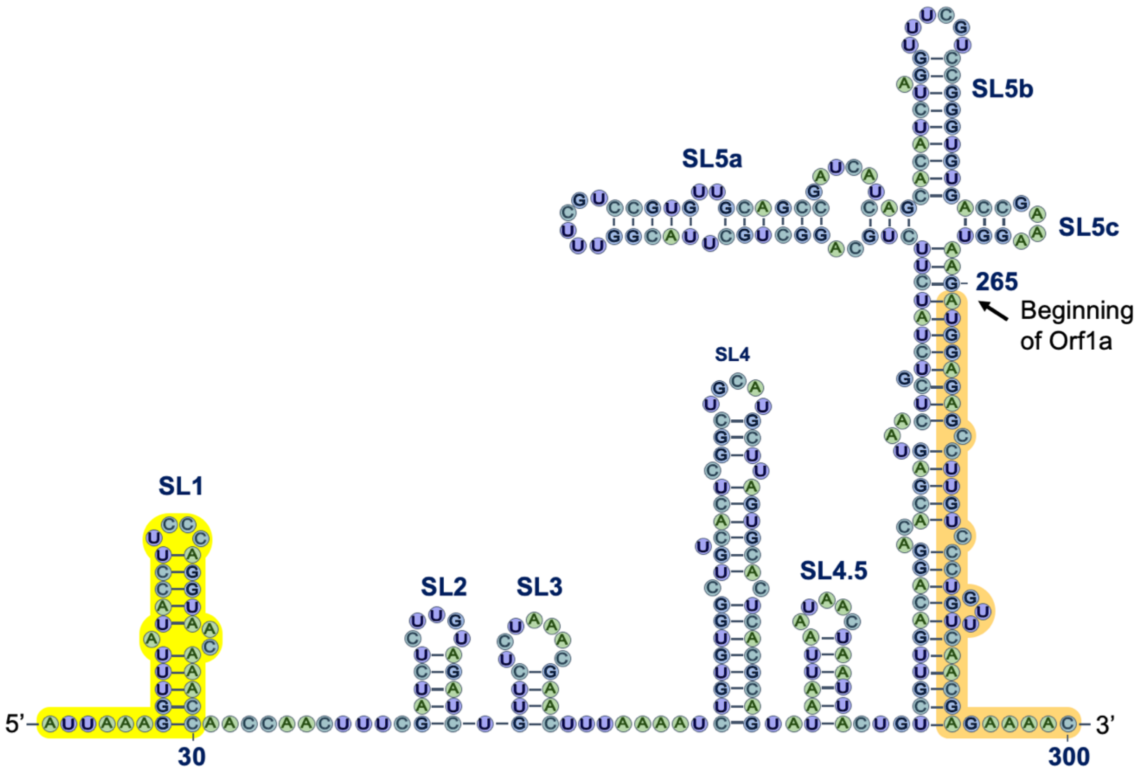

Figure S1. Schematic representation of SARS-CoV-2 5' UTR secondary structure prediction. SL1 highlighted in yellow, and ORF1a highlighted in orange. (SI ref 2)

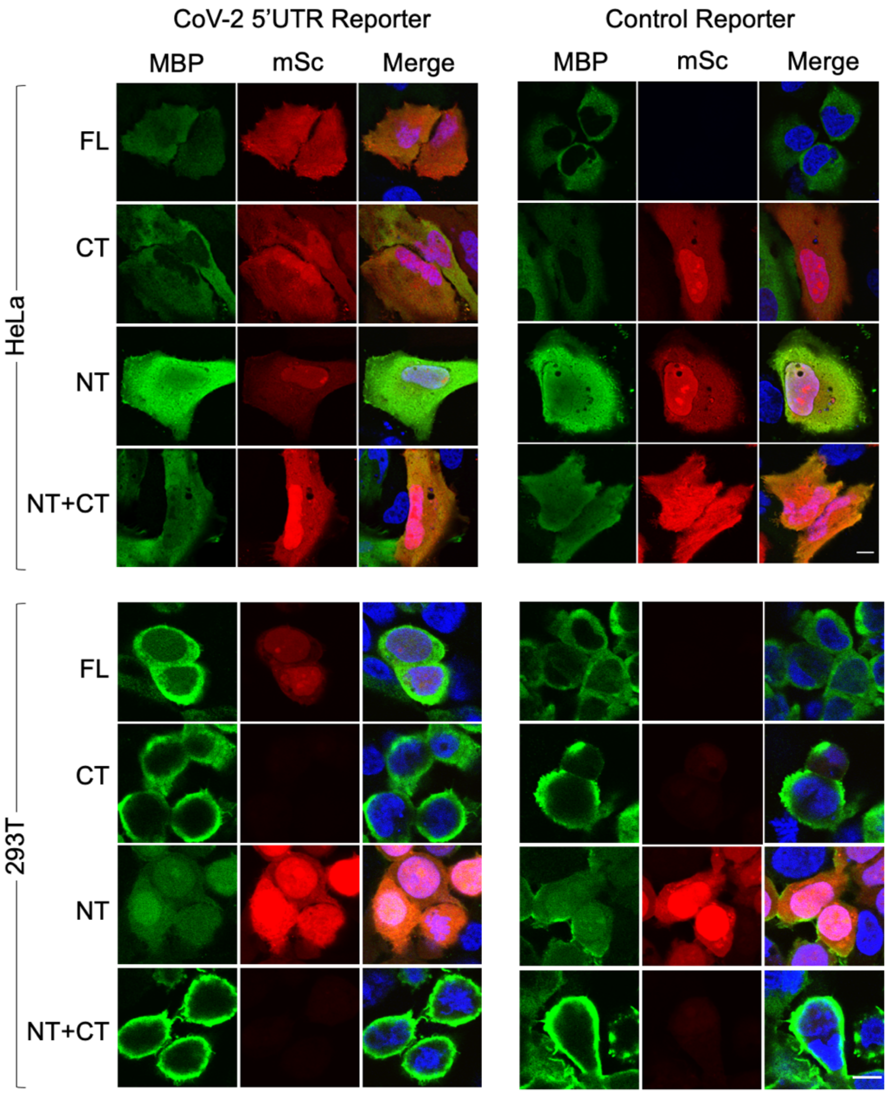

Figure S2. Immunofluorescence of MBP-Nsp1 and in situ fluorescence of reporter activity in HeLa and 293T cells. Cells were stained and visualized for various Nsp1 fragments tagged with MBP. Reporter activity was visualized with in situ mScarlet intensity. In the NT+CT condition, both NT and CT were tagged with MBP. Cells were counterstained with Hoescht 33342 to visualize nuclei.

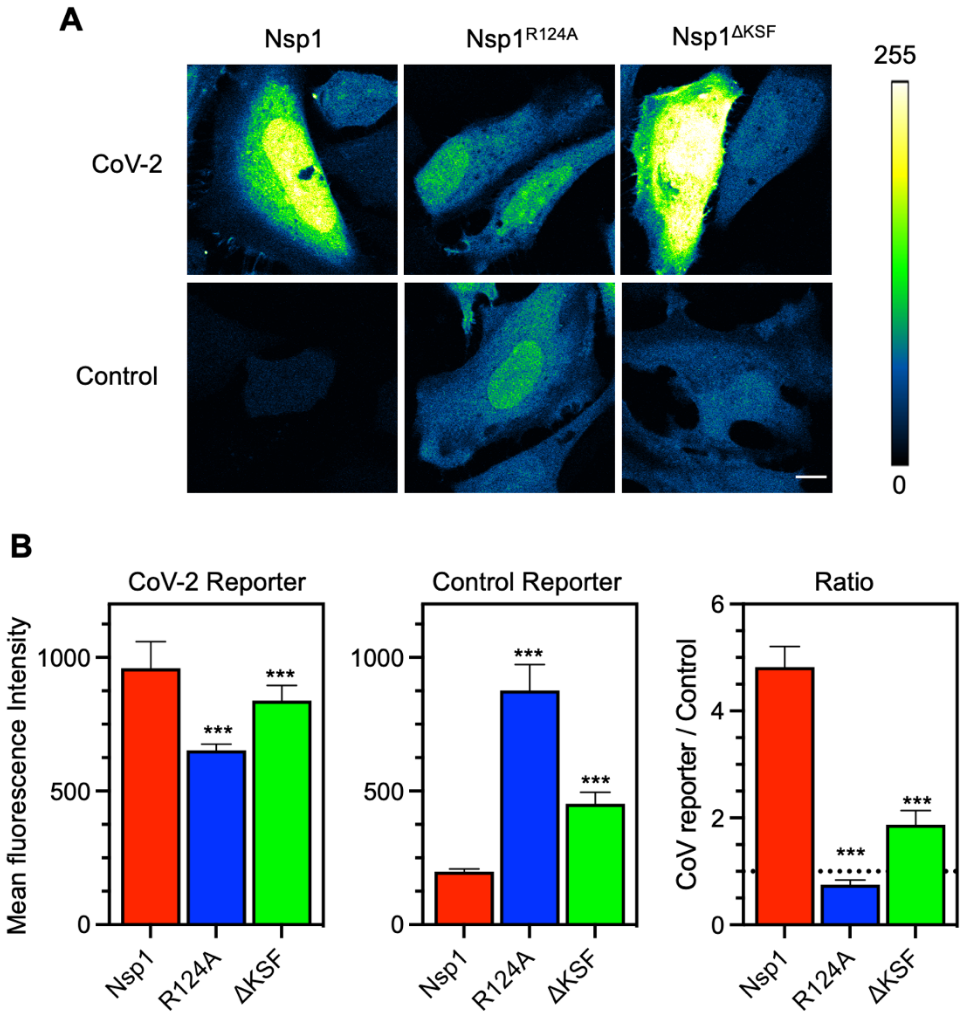

Figure S3. $Nsp1^{R124A}$ and $Nsp1^{\Delta KSF}$ are defective in viral translation selectivity. (A) Images of reporter intensity in HeLa cells transfected with either CoV-2 or control reporter along with various mutants of Nsp1. (B) Mean fluorescence intensity of CoV-2 reporter or control reporter when co-transfected with Nsp1, $Nsp1^{R124A}$, or $Nsp1^{\Delta KSF}$ (left and middle panels) and the ratio between the two reporters (right panel). Error bars represent standard deviation.

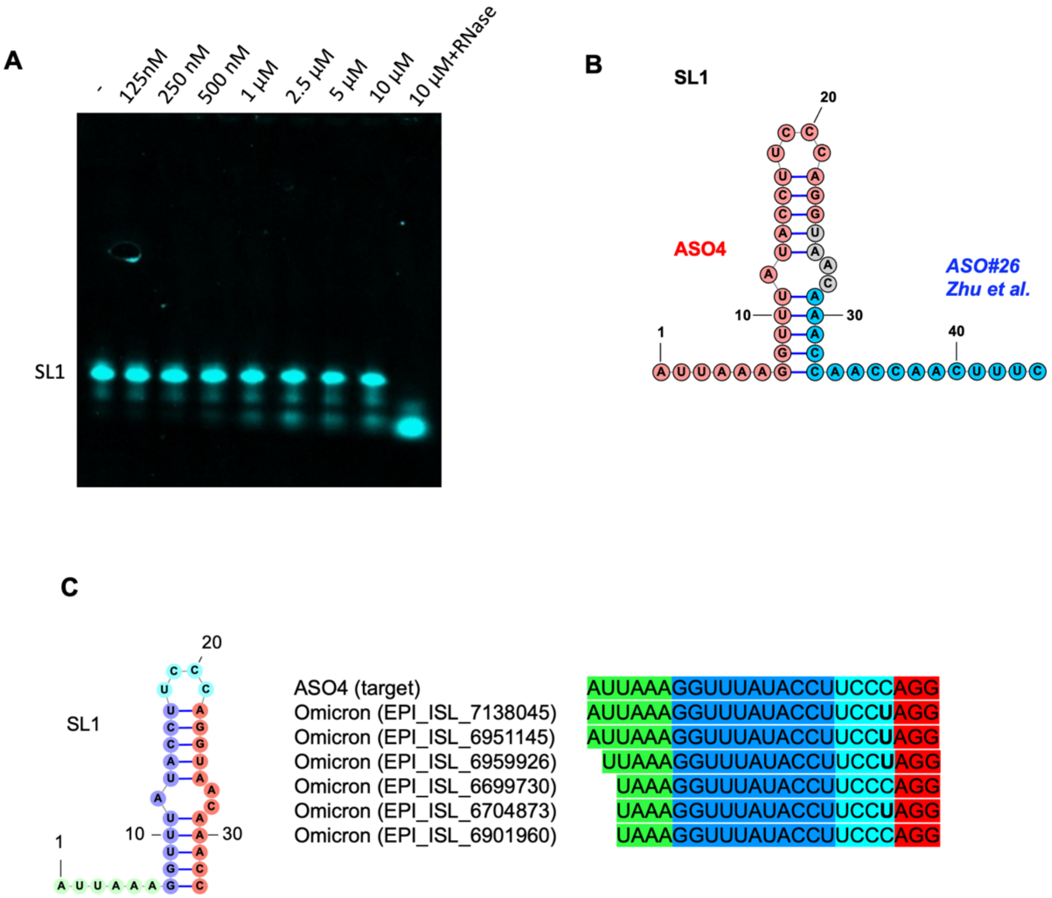

Figure S4. (A) Gel shift assay for SL1-Nsp1 direct interaction. Nsp1 was incubated at various concentrations with 80 nM SL1 for 45 minutes at 37° C (in a buffer composed of 20 mM Tris-HCl 7.5, 100 mM NaCl, 2 mM MgCl2, 1 mM TCEP and 5% Glycerol) and subjected to native polyacrylamide gel electrophoresis**.** (B) ASO4 (pink) and the ASO in (SI ref 4) (blue) target different regions of the 5' UTR. (C) Alignment of ASO4 target sequence with genomic sequences (GISAID accession ID's listed in parenthesis) (SI ref 1) of the SARS-CoV-2 Omicron variant.

Supplemental references

1. S. Elbe, G. Buckland-Merrett, Data, disease and diplomacy: GISAID's innovative contribution to global health. Global Challenges 1, 33-46 (2017).

2. Z. Miao, A. Tidu, G. Eriani, F. Martin, Secondary structure of the SARS-CoV-2 5'-UTR. RNA Biol 18, 447-456 (2021).

3. Z. Yang et al., Tumor-Derived Peptidoglycan Recognition Protein 2 Predicts Survival and Antitumor Immune Responses in Hepatocellular Carcinoma. Hepatology 71, 1626-1642 (2020).

4. C. Zhu et al., An intranasal ASO therapeutic targeting SARS-CoV-2. bioRxiv 10.1101/2021.05.17.444397, 2021.2005.2017.444397 (2021).
